# Associations between lifestyle factors and 2.5-year clinical outcomes differ by time since multiple sclerosis diagnosis

**DOI:** 10.1007/s10072-026-09260-5

**Published:** 2026-08-10

**Authors:** Maggie Yu, George Jelinek, Nupur Nag

**Affiliations:** Neuroepidemiology Unit, Melbourne School of Population and Global Health, The University of Melbourne, Melbourne, VIC Australia

**Keywords:** Multiple sclerosis, Lifestyle, Health outcomes, MS care, Disease duration

## Abstract

**Background:**

Healthy lifestyle factors are associated with better health outcomes in people with multiple sclerosis (pwMS). Whether these associations differ according to time since diagnosis remains unclear. Understanding this may inform optimal timing for lifestyle modification.

**Methods:**

This prospective cohort study included 1,401 pwMS who completed baseline and 2.5-year follow-up surveys from the HOLISM study. Five lifestyle factors were assessed at baseline (high-quality diet, physical activity, smoking status, vitamin D supplementation, meditation). Outcomes included fatigue, depression, and disability. Multivariable log-binomial regression examined associations between individual and multiple lifestyle factors and outcomes stratified by time since diagnosis (≤ 5 vs. > 5 years).

**Results:**

Physical activity showed stronger prospective associations with reduced fatigue, depression, and disability among participants within 5 years of diagnosis than among those with longer duration. Non-smoking was associated with lower disability risk within the ≤ 5-year group. In contrast, high-quality diet was associated with lower depression and disability among participants with > 5-year since diagnosis, though non-significant between-group difference. Vitamin D supplementation and meditation were not associated with outcomes. Meeting 4–5 healthy lifestyle criteria was associated with lower depression risk in both duration groups and with lower fatigue and disability risk in the ≤ 5-year group, with a between-group difference for disability.

**Conclusion:**

Associations between lifestyle factors and MS outcomes may vary across the disease course, suggesting that the duration and timing of lifestyle engagement may influence their potential impact. These findings support further research on stage-specific lifestyle management strategies in MS.

## Introduction

Multiple sclerosis (MS) is a chronic central nervous system disease characterised by early-onset inflammation and neurodegeneration, leading to diverse symptoms and for many people, progressive disability [10]. Symptoms are often present prior to diagnosis, and progression from mild or no disability to moderate disability may be observed within the first two decades from diagnosis [27]. Fatigue and depression are reported in 58% and 33% of people with MS (pwMS), emerging early and persisting across the disease course [32, 39]. As pwMS increasingly seek guidance on lifestyle strategies to optimise medical therapy, understanding when lifestyle modifications may have the greatest impact across the disease course has important implications for clinical management. Early initiation of pharmaceutical treatments is recommended given their effects may change with accumulating damage and declining regenerative capacity [33]. Whether a duration-dependent principle applies to lifestyle behaviours remains largely unknown.

Lifestyle modifications have been shown to be associated with better MS outcomes. High-quality diets are associated with improved fatigue, disability, and QoL [1, 30]. Physical activity may improve fatigue, depressive symptoms, and other health outcomes in people with MS [3]. Smoking is consistently associated with high MS activity, more rapid disability progression, and poor mental health in pwMS, while smoking cessation is linked to slower progression and improved wellbeing [26, 37]. Vitamin D intake has been found to reduce fatigue and improve QoL in some RCTs, while others show no benefits, possibly reflecting lack of initial deficiency or methods of measure [17, 34]. Meditation practice reduces fatigue, depression, anxiety, and stress in pwMS [12]. While most studies show a strong role for diet and physical activity for improved health outcomes, cumulative potential benefits have been observed in pwMS with multiple healthy lifestyle factors [20].

The role of disease duration has rarely been examined in lifestyle research in MS. Many lifestyle studies include participants with a wide range of time since diagnosis (e.g., IQR 2.0–14.0) [13], but disease duration is not consistently reported or incorporated into analyses. Given the progressive nature of MS, it is plausible that associations between lifestyle factors and health outcomes may vary across the disease course [33]. The first five years following diagnosis may represent a particularly important period, as neurological reserve and functional capacity are generally better preserved and disability accumulation is typically less advanced. Consequently, lifestyle factors may have stronger associations with health outcomes earlier in the disease course, when individuals have greater capacity to adopt and maintain behavioural modifications. Supporting this possibility, in a cross-sectional study of 1,083 pwMS, physical activity showed a stronger association with disability among those within 5 years of diagnosis (Nag et al., under review). However, prospective evidence on whether associations differ by disease duration remains lacking.

This study examined associations between five lifestyle factors at baseline and subsequent 2.5-year clinical outcomes in pwMS. Specially, we compared associations among participants within 5 years of diagnosis with those with longer disease duration (> 5 years). Findings may help inform stage-specific lifestyle management strategies in MS.

## Methods

### Study design

This observational cohort study utilised self-reported survey data from the Health Outcomes and Lifestyle In a Sample of people with Multiple Sclerosis (HOLISM) study, described previously [7]. Adults aged ≥ 18 years with self-reported clinician-diagnosed MS completed baseline questionnaires and were invited to complete follow-up questionnaires approximately 2.5 years later. For the present analyses, data from participants who completed both baseline and 2.5-year follow-up surveys were included. Ethical approval was granted by The University of Melbourne Human Research Ethics Committee (ID: 1545102).

### Socio-demographic and clinical variables

Socio-demographic variables included date of birth, sex, education, employment, and partner status; recategorised as binary terms for analysis (Table 1). Clinical variables included body mass index (BMI), calculated using weight (kg)/height(m)^2^ and categorised according to World Health Organisation guidelines [38], underweight and normal were consolidated due to low numbers in the former category. Co-morbidities were queried using the Self-administered Comorbidity Questionnaire [28], reported as total number and dichotomised into ≥ 1 vs. none. MS specific variables included phenotype, dichotomised to non-progressive vs. progressive. Use of any of eleven disease-modifying therapies (DMTs) was consolidated to binary (no/yes). Ongoing symptoms from recent relapse, and use of prescribed anti-fatigue or anti-depression medication, were each queried and reported as binary (no/yes).

**Table 1:** Participant characteristics

	Excluded participants *N *= 1,065	Included participants *N* = 1,401		
	Baseline	Total Baseline	≤ 5 years	> 5 years
MS duration (Median, IQR)	6.4 (2.6–12.4)	5.4 (2.4–11.4)	2.4 (1.4–3.4)	11.4 (7.4–15.5)
N (%)	1,065^a^ (43.2)	1,401^a^ (56.8)	664^b^ (47.4)	737^b^ (52.6)
Socio-demographics				
Age (M, SD)	45.4 (10.5)	45.9 (10.5)	42.3 (10.2)	49.2 (9.7)
Sex				
Male	176 (18.0)	241 (17.3)	123 (18.7)	118 (16.0)
Female	802 (82.0)	1,155 (82.7)	536 (81.3)	619 (84.0)
University education				
No	504 (47.5)	495 (35.6)	214 (32.4)	281 (38.4)
Yes	**556 (52.5)**	**897 (64.4)** ^***^	446 (67.6)	451 (61.6) ^*^
Paid employment				
No	530 (50.2)	570 (40.8)	216 (32.6)	354 (48.2)
Yes	526 (49.8)	828 (59.2)^***^	**447 (67.4)**	**381 (51.8)** ^***^
Partnered				
No	306 (29.4)	334 (24.1)	159 (24.2)	175 (24.0)
Yes	736 (70.6)	1,051 (75.9)	498 (75.8)	553 (76.0)
Clinical measures				
MS phenotype				
Relapsing	652 (73.4)	939 (78.2)	505 (89.9)	434 (67.9)
Progressive	236 (26.6)	262 (21.8)^*^	**57 (10.1)**	**205 (32.1)** ^***^
BMI				
Underweight/normal	538 (51.6)	863 (61.7)	415 (62.5)	448 (61.0)
Overweight	252 (24.2)	304 (21.7) ^***^	136 (20.5)	168 (22.9)
Obese	253 (24.3)	232 (16.6) ^***^	113 (17.0)	119 (16.2)
Comorbidities				
0	561 (52.7)	814 (58.1)	420 (63.3)	394 (53.5)
≥1	504 (47.3)	587 (41.9)^**^	244 (36.7)	343 (46.5)^***^
Recent relapse				
No	751 (70.5)	1,075 (76.7)	489 (73.6)	586 (79.5)
Yes	314 (29.5)	326 (23.3)^**^	175 (26.4)	151 (20.5)
Fatigue (FSS > 5)				
No	397 (45.6)	734 (57.9)	388 (63.8)	346 (52.4)
Yes	**473 (54.4)**	**534 (42.1)** ^***^	**220 (36.2)**	**314 (47.6)** ^***^
Depression (PHQ-2 > 2)				
No	660 (73.0)	1,139 (86.3)	557 (88.7)	582 (84.1)
Yes	**244 (27.0)**	**181 (13.7)** ^***^	71 (11.3)	110 (15.9) ^**^
Taking anti-fatigue medication				
No	939 (88.2)	1,290 (92.1)	633 (95.3)	657 (89.1)
Yes	126 (11.8)	111 (7.9) ^**^	31 (4.7)	80 (10.9) ^**^
Taking anti-depression medication				
No	806 (75.7)	1,158 (82.7)	581 (87.5)	577 (78.3)
Yes	259 (24.3)	243 (17.3)^**^	83 (12.5)	160 (21.7)^**^
Disability (PDDS)				
Normal/mild	476 (50.0)	787 (58.5)	459 (72.2)	328 (46.2)
Moderate/severe	476 (50.0)	559 (41.5)^***^	**177 (27.8)**	**382 (53.8)** ^***^
Lifestyle factors				
High quality diet				
No	705 (66.2)	648 (46.3)	274 (41.3)	374 (50.7)
Yes	**360 (33.8)**	**753 (53.7)** ^***^	390 (58.7)	363 (49.3)^***^
Meditation				
No	673 (73.6)	893 (67.2)	435 (68.7)	458 (65.8)
Yes	241 (26.4)	436 (32.8)^**^	198 (31.3)	238 (34.2)^*^
Physical activity				
No	736 (69.1)	851 (60.7)	380 (57.2)	471 (63.9)
Yes	329 (30.9)	550 (39.3)^***^	284 (42.8)	266 (36.1)^**^
Vitamin D				
No	393 (36.9)	350 (25.0)	141 (21.2)	209 (28.4)
Yes	**672 (63.1)**	**1**,051 (75.0)^***^	523 (78.8)	528 (71.6)^***^
Non-smoker				
No	167 (17.6)	114 (8.5)	51 (8.0)	63 (8.9)
Yes	780 (82.4)	1,227 (91.5)^***^	585 (92.0)	642 (91.1)
Total lifestyle				
0–1	198 (21.7)	156 (11.8)	63 (10.0)	93 (13.5)
2	250 (27.4)	279 (21.1)^*^	120 (19.0)	159 (23.0)
3	244 (26.7)	403 (30.5)^**^	193 (30.5)	210 (30.4)
4–5	**221 (24.2)**	**485 (36.7)** ^***^	256 (40.5)	229 (33.1)^*^

NB: ^a^ percentage of total sample at baseline (*N* = 2466); ^b^ percentage of prospective sample (*N* = 1,401). Results are presented as n (%) unless otherwise stated. T-test and Chi-square tests were conducted to test (1) differences between included and excluded sample characteristics and (2) differences between short and long MS duration groups in characteristics at baseline. Bold values have ≥ 10% and *p* < 0.05 differences

*BMI* Body Mass Index, *FSS* Fatigue Severity Scale, *PDDS* Patient-Determined Disease Steps, *PHQ-2* Patient Health Questionnaire-2, *SD* Standard Deviation

^*^p < 0.05; ^**^p < 0.01; ^***^p < 0.001

### MS duration

MS duration was calculated as the interval between the diagnosis date and the survey submission date. Based on prior research suggesting that early disease stages may represent a critical period for intervention [33], and to ensure adequate sample sizes for comparison, MS duration was categorised as ≤ 5 years and > 5 years from diagnosis.

### Lifestyle factors

Diet was assessed using a modified Diet Habits Questionnaire (DHQ; [19], and high-quality defined as DHQ> median(80). Meditation, queried on frequency (days/week) and duration (min/session) in previous 12 months, was categorised as ≥once/week (no/yes). Physical activity was assessed using the International Physical Activity Questionnaire-Short Form (IPAQ-SF; [2], querying 7-day recall of frequency (days/week) and duration (min) of walking, moderate and vigorous physical activity. Examples provided within the questionnaire included heavy lifting, aerobics, fast bicycling, carrying light loads, and doubles tennis. Participants were classified as physically active if they reported engaging in any intensity of physical activity for ≥ 30 min/day/≥3 times/week. Non-smoking, queried as never, ex, or current smoker, and dichotomised as smoking (current) vs. non-smoking (never/ex). Vitamin D exposure was queried as daily supplement consumed (international units (IU) and dichotomised as ≥ 5,000IU/day (no/yes). Multi-lifestyle variable was derived by summing the “yes” responses for each preceding five lifestyle factors, categorised to ≤ 1, 2, 3 and 4–5 factors based on prior findings [20].

### Health outcomes

Fatigue was queried by Fatigue Severity Scale (FSS), with average mean score of FSS > 5 defined as clinically significant [16]. Depression was assessed using the Patient Health Questionnaire (PHQ)−9 at 2.5-year follow-up and PHQ-2, at baseline. PHQ-2 comprises two items from the PHQ-9 [15]. For prospective analyses, we defined depressive symptoms as PHQ-9 ≥ 10, a threshold with strong psychometric support and MS-specific validation [14, 23]. Disability was assessed using the Patient-Determined Disease Steps (PDDS), a validated patient-reported measure of disability in MS, with PDDS > 3 representing moderate/severe disability [9].

### Statistical analysis

Participant characteristics are presented as mean ± standard deviation (SD) for continuous variables and as frequencies and percentages for categorical variables. To assess attrition bias, baseline characteristics were compared between included and excluded participants using t-tests for continuous variables and chi-square tests for categorical variables.

Cross-sectional associations between baseline lifestyle factors and clinical outcomes were examined using multivariable log-binomial regression models, estimating adjusted prevalence ratios (PRs) with 95% confidence intervals (CIs). For prospective analyses, baseline lifestyle factors were treated as exposures and examined in relation to clinical outcomes assessed 2.5-years later. Associations were estimated using adjusted risk ratios (RRs) and 95% CIs, controlling for baseline outcome values. Effect modification by MS duration was assessed by including interaction terms between MS duration and individual lifestyle factors, as well as the total lifestyle score.

All models were adjusted for age, sex, MS phenotype, recent symptoms due to relapse, education and employment. Additional covariates included disability (for fatigue and depression models); comorbidities and DMT use (for disability models); BMI status and antidepressants (for depression models), and anti-fatigue medications (for fatigue models). Prospective models additionally adjusted for baseline outcome values and recent relapse symptoms at 2.5-year follow-up.

Covariates were selected based on prior analyses of the HOLISM cohort and relevant literature [5, 20]. Baseline variables associated with attrition were additionally assessed in stepwise analyses as potential covariates for multivariable models. Lifestyle factors were mutually adjusted to estimate independent associations.

All data analyses were conducted using Stata version 18.0 (StataCorp LLC, College Station, TX, USA).

## Results

### Participant characteristics

Of the 2,466 pwMS at baseline, 1,401 (57%) completed the 2.5-year follow-up survey and were included in the current study (Table 1). Compared to the study sample, excluded participants differed significantly in most socio-demographic and clinical factors, except for age, sex, and partner status. Material differences (> 10%) were observed for education (64% vs. 53%), fatigue (42% vs. 54%), and depression (14% vs. 27%). Significant differences were observed in all lifestyle factors, with material differences in diet (54% vs. 34%), vitamin D supplementation (75% vs. 63%), and engagement in 4–5 healthy lifestyle factors (37% vs. 24%).

Of the study population, most participants were female, partnered, and had a normal BMI. 47% were within or at 5 years since diagnosis, with an average of 2.4 years since diagnosis, and 53% above 5 years since diagnosis, averaging 12.5 years. At baseline, compared to pwMS within 5 years of diagnosis, those with longer duration were less likely to hold a university degree or be employed, and were more likely to have progressive phenotype, report ≥ 1 comorbidity, and experience fatigue, depression, and moderate/severe disability. Material differences were observed in paid employment (52% vs. 67%), progressive phenotype (32% vs. 10%), fatigue (48% vs. 36%), and moderate/severe disability (54% vs. 28%). Participants with longer disease duration were also less likely to meet criteria for high-quality diet, physical activity, and vitamin D supplementation, whereas meditation was slightly more common and non-smoking was similar; however, none of these lifestyle-factor differences exceeded 10%.

### Associations between lifestyle factors and health outcomes

Of five lifestyle factors assessed, high-quality diet, physical activity, and non-smoking were associated with health outcomes (Tables 2 and 3). Cross-sectionally, high-quality diet was associated with lower fatigue and disability in both groups, and lower depression only in the > 5-year group. For clinical outcomes assessed 2.5-years later, high-quality diet remained associated with lower depression (RR = 0.65; 95%CI 0.45–0.94) and lower disability (RR = 0.89; 95%CI 0.83–0.97) in the > 5-year group, with no between-group differences.

**Table 2:**
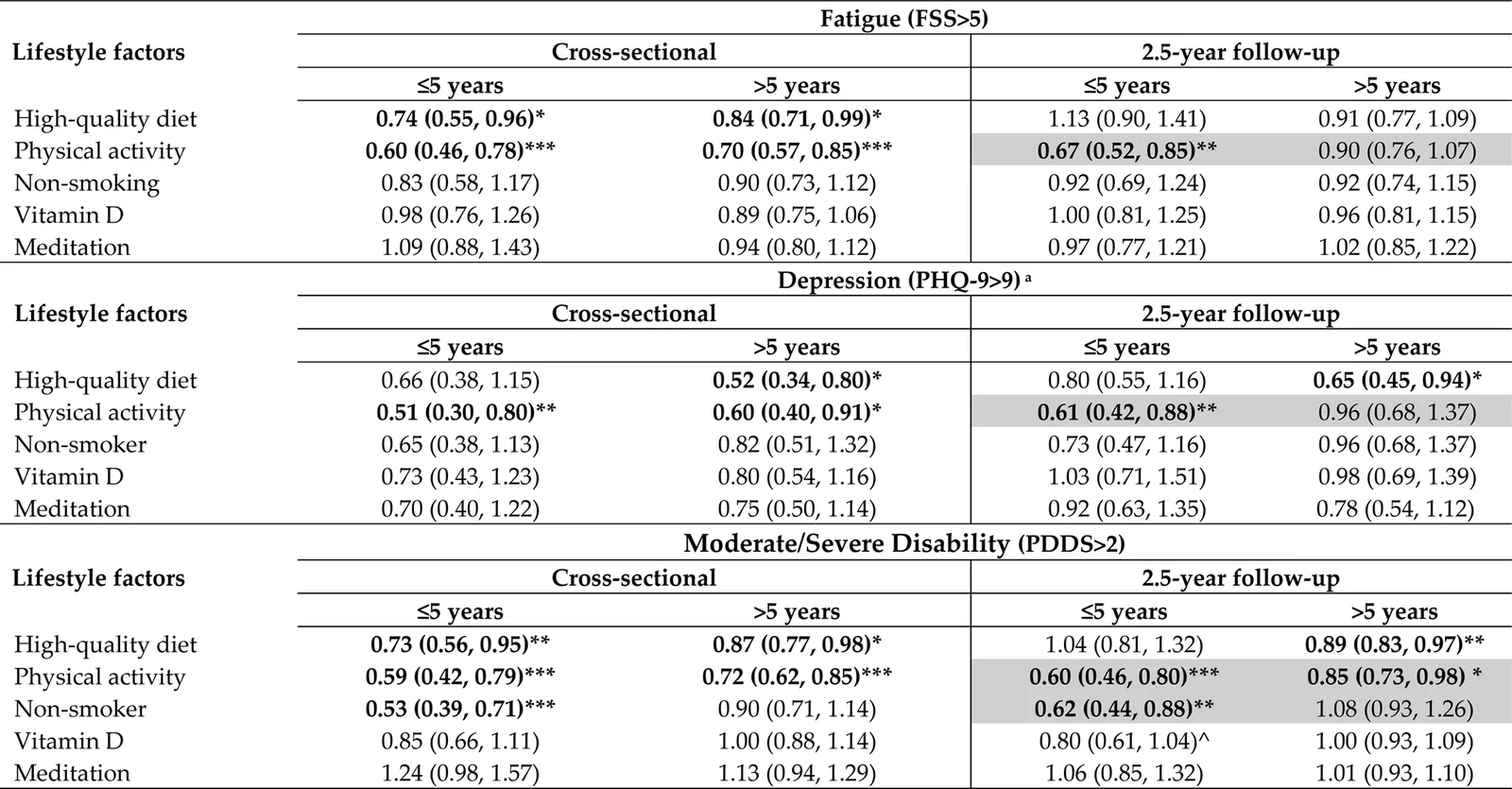
Associations between individual lifestyle factors and health outcomes by MS duration

*PHQ * Patient Health Questionnaire, *FSS * Fatigue Severity Scale, *PDDS * Patient-Determined Disease Steps

Significance notation: ^*p*<0.10; *p<0.05; **p<0.01; ***p<0.001. Grey-highlighted cells indicate significant group differences between the ≤5-year and >5-year MS-duration groups (*p*<0.05)

^a^PHQ-2 was used for cross-sectional analyses at baseline, as PHQ-9 was not available. Multivariable models were adjusted for other lifestyle behaviours, age, sex, MS phenotype, relapse, education, employment, and disability (for fatigue and depression models), comorbidities (for disability), BMI status (for depression), use of DMTs (for disability), anti-fatigue medications (for fatigue), and antidepressants (for depression). Prospective analyses examined associations between baseline lifestyle factors and subsequent 2.5-year clinical outcomes were further adjusted for baseline outcome values and relapse at 2.5-year follow-up

**Table 3:**
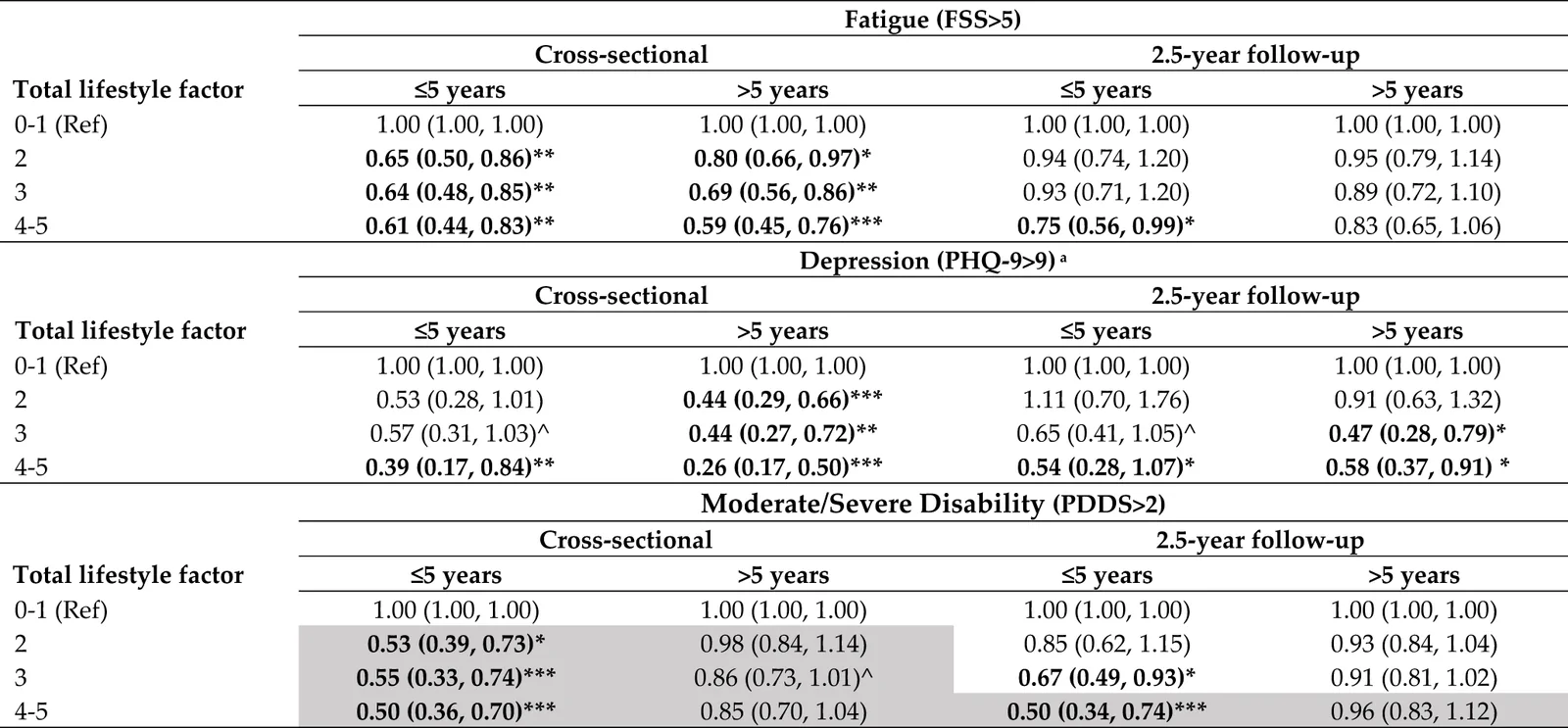
Associations between total lifestyle factors and health outcomes by MS duration

*PHQ * Patient Health Questionnaire, *FSS * Fatigue Severity Scale, *PDDS * Patient-Determined Disease Steps

^a^ PHQ-2 was used for cross-sectional analyses at baseline, as PHQ-9 was not available. Multivariable models were adjusted for age, sex, MS phenotype, relapse, education, employment, and disability (for fatigue and depression models), comorbidities (for disability), BMI status (for depression), use of DMTs (for disability), anti-fatigue medications (for fatigue), and antidepressants (for depression). Prospective analyses examined associations between baseline lifestyle factors and clinical outcomes assessed 2.5 years later and were further adjusted for baseline outcome values and relapse at 2.5-year follow-up

Significance notation: ^*p*<0.10; *p<0.05; **p<0.01; ***p<0.001. Grey-highlighted cells indicate significant group differences between the ≤5-year and >5-year MS-duration groups (*p*<0.05)

Physical activity was cross-sectionally associated with lower risk of all three outcomes in both groups. Prospectively, associations retained for fatigue (RR = 0.67; 95% CI 0.52–0.85) and depression (RR = 0.61; 95% CI 0.42–0.88) only in the ≤ 5-year group, with significant between-group differences (*p* < 0.05). For disability, prospective associations were observed in both duration groups but were stronger in the ≤ 5-year group (RR = 0.60; 95%CI 0.46–0.80) than in the > 5-year group (RR = 0.85; 95%CI 0.73–0.98), with a significant between-group difference (*p*-interaction < 0.05).

Non-smoking was cross-sectionally associated with lower disability in the ≤ 5-year group and remained significant prospectively (RR = 0.62; 95% CI: 0.44–0.88), with evidence of a between group difference (*p*-interaction < 0.05).

No significant associations were observed for vitamin D supplementation and meditation.

### Multi-lifestyle associations with health outcomes

Engagement with multiple healthy lifestyle factors was associated with lower risk of fatigue, depression, and disability (Table 4). Cross-sectionally, multi-lifestyle engagement was associated with lower fatigue and depression in both duration groups. For disability, a clear dose–response relationship was observed in the ≤ 5-year group, with significant between-group differences across levels of lifestyle engagement (*p*-interaction < 0.05).

**Table 4:**
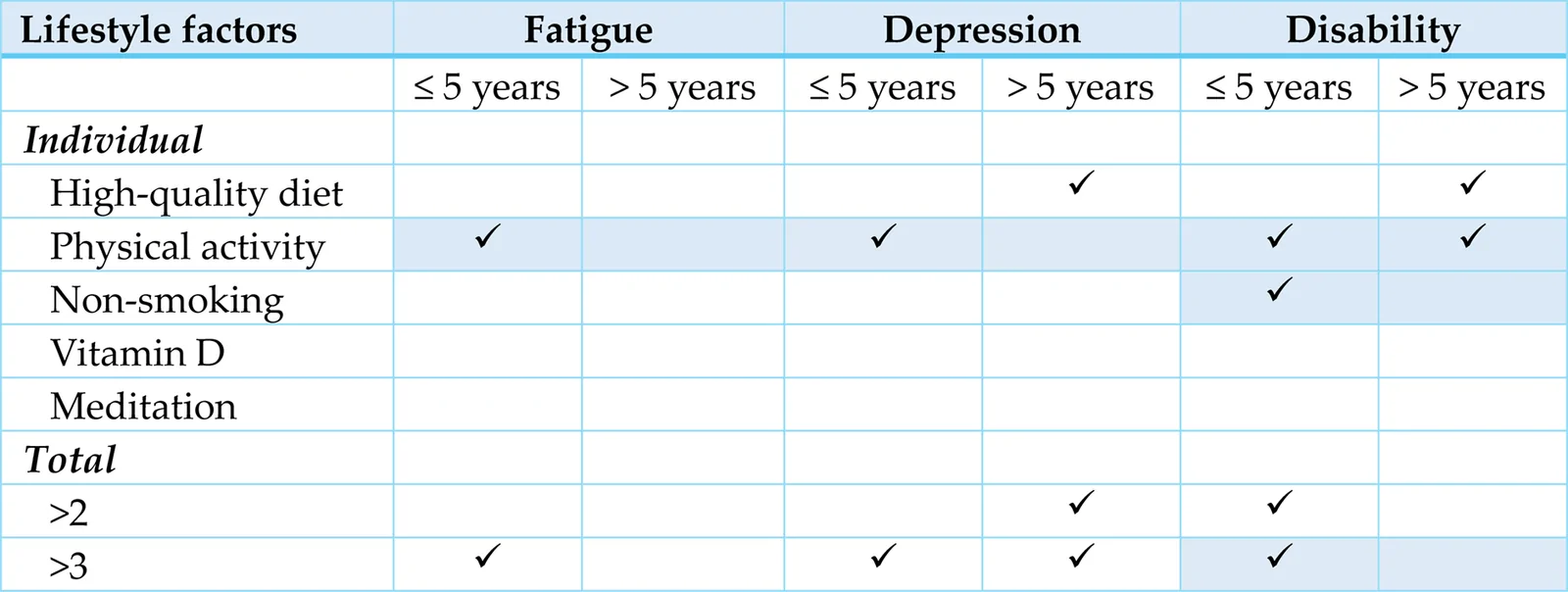
Associations between lifestyle factors and health outcomes

✓ significant prospective associations between lifestyle factors and PROMs (*p* < 0.05); Highlighted cells indicate significant group differences between the ≤5-year and >5-year MS-duration groups (*p*<0.05)

Associations between baseline lifestyle engagement and clinical outcomes at 2.5-year follow-up showed that engagement in 4–5 healthy lifestyle behaviours was associated with a reduced risk of fatigue in the ≤ 5-year group (RR = 0.75; 95% CI 0.56–0.99), with no between-group difference. For depression, prospective associations were observed for 3 behaviours in the > 5-year group, and for 4–5 behaviours in both groups, without significant between-group differences. For disability, prospective associations were observed for ≥ 3 healthy behaviours in the ≤ 5-year group (RR = 0.50; 95% CI 0.34–0.74), and the between-group difference was significant only for engagement in 4–5 behaviours (*p*-interaction < 0.05).

## Discussion

In this prospective cohort study, associations between healthy lifestyle factors and MS clinical outcomes differed according to time since diagnosis. Physical activity showed stronger prospective associations with lower fatigue, depression, and disability, among participants within 5 years of diagnosis, while non-smoking was associated with lower disability primarily in this early disease group. In contrast, high diet quality showed clear prospective associations with depression and disability among participants with longer disease duration, though without significant between-group differences. Engagement with multiple healthy lifestyle factors was associated with reduced depression risk across both groups, but with reduced fatigue and disability primarily within the ≤ 5-year group.

The study cohort was predominantly female and university educated, with approximately half of participants within 5 years of diagnosis. MS duration in the analytic sample was broad (median 5.4 years, IQR 2.4–11.4), consistent with the heterogeneous disease-duration profiles commonly seen in observational MS studies [13, 21]. Overall, most participants met the non-smoking and vitamin D supplementation criteria, and the majority met at least two healthy lifestyle factor criteria. Compared with those lost to follow-up, retained participants were more likely to meet healthy diet and vitamin D criteria and less likely to report fatigue or depression. Although this suggests potential healthy participant bias and may limit generalisability, it also indicates that lifestyle modifications were acceptable and commonly adopted for MS self-management. As expected, participants with > 5 years since diagnosis were less likely to be employed and more likely to have progressive MS, fatigue, and moderate/severe disability than those within ≤ 5 years. These characteristics are consistent with the progressive nature of MS and may partly explain why associations between lifestyle factors and clinical outcomes differed according to disease duration.

High-quality diet has been associated with lower disability, depression, and fatigue in pwMS [5, 20]. These potential benefits may reflect anti-inflammatory, neuroimmune, metabolic, vascular, and gut–brain mechanisms [31]. In the current study, high-quality diet was prospectively associated with reduced depression and disability only in the > 5-year group, although between-group differences were non-significant. In keeping with the long term benefits of healthy diet seen in other common western chronic diseases, this may suggest that benefits of diet require sustained adherence before measurable effects on outcomes emerge, as we previously reported [40]. In the earlier study, 2.5-year adherence to a high-quality diet was required to observe lower fatigue, depression, and disability, whereas shorter adherence was not associated with potential benefit. Similarly, maintaining a high healthy-diet score across 11-years was associated with lower depression over the subsequent 13 years [24].

Physical activity has consistently been associated with benefits on fatigue, mobility, and depression for pwMS [6], and is recommended as a part of standard care [11]. Only one earlier study examined differences across MS disease course [18]; in this RCT (*N* = 40), greater mobility gains from walking were observed among pwMS diagnosed within vs. > 1 year. Similarly, we observed prospective associations between regular physical activity and lower fatigue and depression only in the ≤ 5-years group, with significant between-group differences, whereas associations with lower disability were observed in both groups but stronger in the ≤ 5 years group. These findings suggest that physical activity may be particularly beneficial earlier in the disease course, while benefits for disability may extend across disease duration. Physical activity may influence MS outcomes through multiple pathways, including reduced neuroinflammation, improved neuroplasticity, enhanced cardiovascular fitness, preservation of muscle strength, and improved mobility and balance. These effects may be particularly relevant early in the disease course, when disability is less advanced and opportunities for intervention may be greatest [3, 33]. In addition, we previously found that participants who self-reported impaired mobility as a top symptom important to them, were less likely to meet the criteria for physical activity [8].

Smoking is a key MS risk factor that increases neuroinflammation and is associated with greater disability and faster disability progression [26]. In the current study, non-smoking was associated with reduced disability, more strongly within ≤ 5-year of diagnosis, supporting smoking cessation early in the disease course. While prospective associations between smoking status and fatigue and depression have been reported in a systematic review of 12 studies [36], we did not observe these associations in this population, possibly due to the small proportion of current smokers (< 10%) and the consolidation of ex-smokers (38%) with never-smokers (53%).

Interventional studies in MS have reported beneficial effects of high-dose vitamin D on disease activity or fatigue, but findings are inconsistent and appear most evident in participants who are vitamin D–deficient at baseline or who achieve substantial increases in serum 25(OH)D [29]. We did not observe significant associations between daily 5,000 IU vitamin D supplementation and fatigue, depression, or disability. This may reflect characteristics of the study sample, in which vitamin D supplementation was very common and baseline vitamin D status was not available for consideration.

Meditation may affect MS outcomes through stress-regulatory, inflammatory, and neuroimmune pathways [35]. In the current study, no significant associations were observed between meditation and outcomes. Interventional studies have reported improvements in fatigue, although these typically involve structured programs delivered over several weeks [4]. In our recent prospective analysis of the 2.5- to 7.5-year follow-up cohort, meditation was associated with improved depression, but not fatigue [20]. These differences may partly reflect cohort characteristics and measurement limitations. Meditation has many forms of practice and benefits may differ by modality as well as individual skill and mental readiness. Our measure used a single self-reported item referring to any meditation practice in the prior 12 months and likely captured a heterogeneous mix of informal or inconsistent practice. More structured and sustained practice may be required before measurable benefits emerge.

Engagement with multiple healthy lifestyle factors has cumulative beneficial associations for fatigue, depression, and disability [20]. These factors may influence biological processes through epigenetic mechanisms affecting inflammation, metabolism, and stress regulation [22]. The current findings also show engagement in multiple healthy lifestyle factors are associated with better outcomes, with the stronger associations observed in ≤ 5-year group for disability.

## Strengths and limitations

Limitations are acknowledged in that the study population represented 57% of the baseline cohort, with evidence of attrition and healthy participant bias, which may limit generalisability. Second, adherence, duration, and frequency of lifestyle practices before and after diagnosis were not captured, limiting inference about timing and dose. Several lifestyle factors were assessed using broad measures designed to capture overall engagement rather than specific modalities. For example, physical activity was classified according to activity intensity and frequency, diet quality reflected overall dietary patterns rather than specific dietary approaches, and meditation was assessed without information on format or context. Third, lifestyle behaviours may have changed during the 2.5-year follow-up period; therefore, baseline measures may not fully reflect behaviours throughout follow-up. As such, these analyses do not provide information on the effects of sustained lifestyle engagement or behavioural changes over time. Finally, clinical characteristics (e.g., MS phenotype), lifestyle factors, and outcomes were self-reported, including disability assessed using the PDDS rather than clinician-assessed EDSS, introducing potential misclassification.

However, strengths of this study include its 2.5-year prospective design and a large population of pwMS. To our knowledge, this is the first analysis to assess prospective associations between individual and multimodal lifestyle factors and clinical outcomes by time since diagnosis demonstrating longevity of benefits. Models were mutually adjusted for other lifestyle behaviours and providing an estimate of each factor’s potentially independent effect. By additionally adjusting for relapse history, MS phenotype, disability level, and medication use, we reduced confounding by disease severity and treatment.

Our findings support a “time matters” perspective, in which associations between lifestyle factors and MS outcomes may differ across the disease course and some benefits may depend on sustained adherence [25, 40]. Reverse causality cannot be excluded, as individuals with greater disability may be less able to engage in certain lifestyle behaviours, particularly physical activity [8]. Together, these findings support a personalised, stage-appropriate approach in which pwMS are supported to prioritise different healthy lifestyle behaviours across the disease course.

## Conclusion

As MS progresses, increasing symptom burden may affect adherence to lifestyle practices and their benefits. These findings suggest that associations between lifestyle factors and MS outcomes may vary across the disease course. Encouraging and supporting a healthy lifestyle through shared decision-making, realistic goal-setting, and regular review may therefore form an important component of comprehensive MS management. Early engagement in behaviours such as physical activity and smoking cessation may offer greater potential to influence clinical outcomes during the initial years following diagnosis.

## Data Availability

Data available from NN upon reasonable request.
